# Effect of model updating strategies on the performance of prevalent diabetes risk prediction models in a mixed-ancestry population of South Africa

**DOI:** 10.1371/journal.pone.0211528

**Published:** 2019-02-07

**Authors:** Katya L. Masconi, Tandi E. Matsha, Rajiv T. Erasmus, Andre P. Kengne

**Affiliations:** 1 Division of Chemical Pathology, Faculty of Health Sciences, National Health Laboratory Service (NHLS) and University of Stellenbosch, Cape Town, South Africa; 2 Non-Communicable Diseases Research Unit, South African Medical Research Council, Cape Town, South Africa; 3 Department of Biomedical Technology, Faculty of Health and Wellness Sciences, Cape Peninsula University of Technology, Cape Town, South Africa; 4 Department of Medicine, University of Cape Town, Cape Town, South Africa; Liverpool School of Tropical Medicine, UNITED KINGDOM

## Abstract

**Background:**

Prediction model updating methods are aimed at improving the prediction performance of a model in a new setting. This study sought to critically assess the impact of updating techniques when applying existent prevalent diabetes prediction models to a population different to the one in which they were developed, evaluating the performance in the mixed-ancestry population of South Africa.

**Methods:**

The study sample consisted of 1256 mixed-ancestry individuals from the Cape Town Bellville-South cohort, of which 173 were excluded due to previously diagnosed diabetes and 162 individuals had undiagnosed diabetes. The primary outcome, undiagnosed diabetes, was based on an oral glucose tolerance test. Model updating techniques and prediction models were identified via recent systematic reviews. Model performance was assessed using the C-statistic and expected/observed (E/O) events rates ratio.

**Results:**

Intercept adjustment and logistic calibration improved calibration across all five models (Cambridge, Kuwaiti, Omani, Rotterdam and Simplified Finnish diabetes risk models). This was improved further by model revision, where likelihood ratio tests showed that the effect of body mass index, waist circumference and family history of diabetes required additional adjustment (Omani, Rotterdam and Finnish models). However, discrimination was poor following internal validation of these models. Re-estimation of the regression coefficients did not increase performance, while the addition of new variables resulted in the highest discriminatory and calibration performance combination for the models it was undertaken in.

**Conclusions:**

While the discriminatory performance of the original existent models during external validation were higher, calibration was poor. The highest performing models, based on discrimination and calibration, were the Omani diabetes model following model revision, and the Cambridge diabetes risk model following the addition of waist circumference as a predictor. However, while more extensive methods incorporating development population information were superior over simpler methods, the increase in model performance was not great enough for recommendation.

## Introduction

Predictive performance is often decreased when a model is tested in a population different to that in which the model was developed. To limit the number of models redeveloped in smaller datasets due to poor performance of existent models, updating methods aim to improve the prediction performance of a model in a new setting [1]. The updating of an existent model is encouraged as it allows for the information captured during the development of the model to be incorporated with the characteristics of the validation population [1–5].

Several updating methods are available in statistical literature [1, 4–6]. These methods vary in the extent to which the model is adjusted, and range from simple recalibration, in which only the intercept of the model may be updated, to more extensive updating, where all the model parameters are re-estimated and new predictors are considered. There is no advocated method to use, however there are limitations to both the most commonly used approach, namely simple intercept correction, which does not account for the difference in strength of the individual variables in the validation population, and the re-estimation of the regression coefficients, which replaces unbiased estimates and fits the model with the validation outcome prevalence, which can be unreliable [6]. Updating methods are, however, not a remedy against poorly conceived and underpowered prediction research, nor do they guarantee complete bridging of the gaps due to large differences between development and validation datasets. How these methods alter the performance of existent prevalent diabetes risk prediction models during the validation in empirical data has not yet been investigated.

In this study, we externally validate existent models, developed in different populations, and applied the updating methods presented by Janssen *et al* [6], adapted from Steyerberg *et al* [7], in a dataset from South Africa, where population specific diabetes risk prediction models are not available. The performance was assessed to determine if they can be improved enough to allow recommendation for use.

## Materials and methods

### Study population

Details of the study design and recruitment of the dataset that served as the basis for all updating methods implementation, are described in more detail elsewhere [8]. Briefly, Bellville-South is located within the Northern suburbs of Cape Town, South Africa and is traditionally a mixed-ancestry township formed in the late 1950s. The target population for this study were subjects between the ages of 35 and 65 years of age and their number was estimated to be 6 500 in the 2001 population census [9]. The data was collected during January 2008 to March 2009, and community authorities requested that participants outside the random selection area should benefit from the study. Recruited subjects gave written consent and were visited by the recruitment team the evening before participation to be reminded of all the survey instructions. The study was approved by the Ethics Committee of the Cape Peninsula University of Technology and Stellenbosch University.

### Predictors

A questionnaire was administered to obtain information on lifestyle factors, such as smoking and alcohol consumption, physical activity, diet, family history of diabetes mellitus, and demographics. A detailed drug history was obtained through interviews, the examination of the clinic cards, as well as the recording of drugs that participants brought to the study site. Clinical measurements included height, weight, hip and waist circumferences, body fat measurements and blood pressure.

### Outcome

All participants, except self-reported diabetic subjects, confirmed by either medical card record or drugs in use, had blood taken for fasting blood glucose and underwent a 75 g oral glucose tolerance test (OGTT), as prescribed by the World Health Organisation (WHO). Diabetes was diagnosed according to the WHO 2006 criteria [10].

### Identification of prevalent diabetes prediction models

Existing prediction models were obtained from a systematic review by Brown *et al*, 2012 [11]. Models met the criteria for model selection for this paper if they were developed to predict the presence of undiagnosed diabetes, and used only variables that were measured in the Bellville South study. We focused on models developed from non-invasively measured predictors. Therefore, the models retained were as follows: Cambridge Risk model [12], Kuwaiti Risk model [13], Omani Diabetes Risk model [14], Rotterdam Predictive model 1 [15] and the simplified Finnish Diabetes Risk model [16]. Model characteristics, formulas and development performance are available elsewhere [17]. All models included age as a predictor, while a range of other predictors were included in varying combinations in the models, namely sex, body mass index (BMI), use of antihypertensive medication, family history of diabetes, waist circumference, past or current smoking and the use of corticosteroids.

### Statistical methods

#### Analysis of missing data

The proportion of missing data for each predictor was determined, with family history having the most missing data [mother (25.1%), father (24.9%), sister (25.0%), and brother (25.1%)]. The remaining predictors had a missing proportion of less than 5%, except smoking status (6.1%). During the comparison of several imputation methods in this dataset on the effect on model performance, simple imputation (mean or mode substitution) allowed for a similar predictive performance of a risk prediction model, when compared to more complex imputation methods, and was therefore used to handle missing data in this study, prior to the implementation of any updating methods [18].

#### Updating methods

Updating methods ranged in the extent to which both the original model was altered and in the requirement of the development and validation datasets [6, 7, 19]. This study naturally did not have access to the development datasets of the selected prevalent diabetes risk prediction models, therefore excluding updating methods that required the merging of both development and validation datasets. The selected models were initially run without adjustment, Method 0, termed the ‘reference method’. These were run in the full dataset, as an external validation of these models, and to which all updating methods were compared, to determine whether an updated formula could offer better validated predictive performance. The updating techniques, explained by Janssen *et al* [6], were used to update the prevalent diabetes risk prediction models in this study. The data was split, with two-thirds of the data used for the implementation of the updating methods, and the final third used for internal validation of the newly updated models. Methods 1 and 2 refer to recalibration. Method 1 updated only the intercept using a correction factor to correct for the difference in disease prevalence between the development and validation population, termed ‘intercept adjustment.’ Method 2 updated both the intercept and the regression coefficients of the variables using the intercept and calibration slope from Method 1 respectively, termed ‘logistic calibration.’ Method 3–5 were more comprehensive revision methods. Method 3, termed ‘revision,’ tested whether the effect of each variable is different in the updating dataset, following the calibration of Method 2. Predictors were individually added as an offset, calculating a deviation from the recalibrated regression coefficient based on Method 2. Likelihood ratio tests were used to test whether this deviation has added predictive value. The same predictors and regression coefficients from the original model were used, and the deviation of the predictors with statistically significant differences was added to the linear predictor from Method 2. Method 4, termed ‘re-estimation,’ was the complete re-estimation of the intercept and the regression coefficients, fitting the predictors from the original models in the validation dataset. Finally, the effect of additional predictors on each model was considered (Method 5). The following predictors were offered to each model univariately, following the same methodology as Method 3 to test their statistical importance: Cambridge risk model: systolic blood pressure, diastolic blood pressure, highest education status (categorical: primary school, high school, university), use of lipid lowering drugs, drinking status (categorical: never, ex and current) and waist circumference (≥94cm for men, ≥80 for women); Kuwaiti risk model: sex, BMI (categorical: 25 kg/m^2^ ≤ BMI < 30 kg/m^2^ and BMI ≥ 30 kg/m^2^), systolic blood pressure, diastolic blood pressure, education status, use of lipid lowering drugs, use of corticosteroids, (categorical: never, ex and current) and drinking status; Omani diabetes risk model: sex, education status, use of lipid lowering drugs, use of corticosteroids, smoking status and drinking status; Rotterdam predictive model: systolic blood pressure, diastolic blood pressure, education status, use of lipid lowering drugs, use of corticosteroids, family history of diabetes, smoking status, drinking status and waist circumference; and simplified Finnish diabetes risk model: sex, systolic blood pressure, diastolic blood pressure, education status, use of lipid lowering drugs, use of corticosteroids, family history of diabetes, smoking status and drinking status. Additional predictors were not offered to the models if they was already included in some form. For methods 3–5, parameterwise model shrinkage was undertaken to adjust for possible overfit. All analyses were conducted using the R software for statistical computing.

#### Model development

As a reference for the comparison of the model performance of the model produced from each of the updating methods, a model was developed. Backward stepwise selection was used to select the predictors. The predictors made available for selection were sex, age, BMI (categorical: 25 kg/m^2^ ≤ BMI < 30 kg/m^2^ and BMI ≥ 30 kg/m^2^), systolic blood pressure, diastolic blood pressure, waist circumference (≥94cm for men, ≥80 for women), highest education status (categorical: primary school, high school, university), use of hypertensive medication, use of lipid lowering drugs, use of corticosteroids, family history of diabetes, smoking status (categorical: never, ex and current) and drinking status (categorical: never, ex and current). A logistic regression model was fit with the selected predictors, with coefficients shrunk parameterwise.

#### Model performance

The selected models were validated in the overall data using the original structure, without any recalibration. The predicted probability of undiagnosed diabetes for each participant was computed using the baseline measured predictors. The performance was expressed in terms of discrimination and calibration. Discrimination describes the ability of the model’s performance in distinguishing those at a high risk of developing diabetes from those at low risk [20]. The discrimination was assessed and compared using concordance (C) statistic [21].

Calibration describes the agreement between the probability of the outcome of interest as estimated by the model, and the observed outcome frequencies [1]. It was assessed by calibration plots and computation of the expected (E) over observed (O) ratio (E/O); with the 95% confidence intervals calculated assuming a Poisson distribution [22]. We also calculated 1) the Yates slope, which is the difference between mean predicted probability of type 2 diabetes for participants with and without prevalent undiagnosed diabetes, with higher values indicating better performance; and 2) the Brier score, which is the squared difference between predicted probability and actual outcome for each participant with values ranging between 0 for a perfect prediction model and 1 for no match in prediction and outcome [1, 20].

## Results

### Updating dataset

The study sample consisted of 1256 individuals, of whom 173 were excluded due to previously diagnosed diabetes. Of the final 1083 individuals, 329 (30.4%) had missing data, which were imputed using simple imputation. The characteristic profile for the split datasets are described in Table 1. The mean age was 51.9 (14.9) years and a total of 162 (15%) individuals had undiagnosed diabetes. The database included 832 (76.8%) females. A comparison between the training and test datasets only showed a statistically significant difference for diastolic blood pressure.

**Table 1:** Demographic characteristics of the Bellville South cohort, by the training and test datasets.

Variables	Overall (1083)	Training dataset	Test dataset	P-value
Prevalent undiagnosed diabetes (Yes, %)	162 (15.0)	118 (15.6)	44 (13.5)	0.354
Sex (Male, %)	251 (23.2)	169 (22.3)	82 (25.2)	0.174
Age (mean years, SD)	51.9 (14.9)	52.0 (14.9)	51.6 (15.1)	0.691
Body mass index (mean kg/m2, SD)	29.7 (7.0)	29.6 (6.9)	30.0 (7.4)	0.503
Waist circumference (mean cm, SD)	95.8 (15.3)	95.8 (15.2)	95.9 (15.6)	0.919
Systolic blood pressure (mean mmHg, SD)	124.3 (20.0)	123.6 (20.2)	126.1 (19.4)	0.056
Diastolic blood pressure (mean mmHg, SD)	76.0 (12.7)	75.4 (12.8)	77.3 (12.4)	0.026
Use of hypertensive medication (Yes, %)	374 (34.5)	268 (35.4)	106 (32.6)	0.142
Hypertensive status (Yes, %)	817 (75.4)	570 (75.2)	247 (76.0)	0.755
Use of lipid-lowering medication (Yes, %)	40 (3.7)	30 (4.0)	10 (3.1)	0.579
Use of corticosteroids (Yes, %)	12 (1.1)	5 (0.7)	7 (2.2)	0.062
Mother having diabetes (Yes, %)	124 (11.5)	87 (11.5)	37 (11.4)	0.999
Father having diabetes (Yes, %)	61 (5.6)	38 (5.0)	23 (7.1)	0.195
Sister having diabetes (Yes, %)	103 (9.5)	71 (9.4)	32 (9.9)	0.873
Brother having diabetes (Yes, %)	67 (6.2)	49 (6.5)	18 (5.5)	0.631
Alcohol use (Current, %)	272 (25.1)	186 (24.5)	86 (26.5)	0.384
Smoking status (Current, %)	433 (40.0)	304 (40.1)	129 (39.7)	0.847
Education (High School, %)	131 (12.1)	94 (12.4)	37 (11.4)	0.663

SD, standard deviation

### Models parameters

Method 1–3 correction estimates are presented in Table 2, and full model formulas for the original model, method 4 and method 5 are presented in Table 3, all derived in the training dataset. Baseline predicted risks by the Cambridge and Omani diabetes risk models was too low, requiring the intercept to be decreased further during the intercept adjustment (Method 1) (-6.322 to -7.205, and -4.700 to -5.083, respectively), while the predicted risk was too high and the intercept increased for the Kuwaiti, Rotterdam and Simplified Finnish diabetes risk models. Logistic calibration (Method 2) showed additional adjustment to the intercept of all models, increasing the underlying risk, and the correction of the regression coefficients of the original models with the calibration slopes from method 0, showed that all models required the weighting of their predictors to be decreased. The likelihood ratio test results from model revision showed no significantly different effect for any predictor for the Cambridge and Kuwaiti risk models. A number of predictors required adjustment over and above the calibration slope adjustment from method 2 for the Omani diabetes risk model, namely a greater predictive effect of BMI ≥ 30 kg/m^2^ (0.115) and a WC ≥ 94 cm in men and ≥ 80cm in women (0.890) and a lower predictive effect for a parent or sibling having a history of diabetes (-0.253). Only sex needed adjustment in the Rotterdam predictive model (-0.783), while age (45 years ≤ age ≤ 54 years) and the use of hypertensive medication where reduced and increased in the simplified Finnish diabetes risk model, respectively.

**Table 2:** Estimated parameters of the updating methods 1–3.

		Method 1	Method 2	Method 3
Cambridge Diabetes Risk model	Correction factor (1) / Calibration intercept (2–3)	-0.883	- 1.617	^-^
	Calibration slope used for linear predictor correction	-	0.263	-
Kuwaiti Risk model	Correction factor (1) / Calibration intercept (2–3)	0.304	-1.008	-
	Calibration slope used for linear predictor correction	-	0.342	-
Omani Diabetes Risk model	Correction factor (1) / Calibration intercept (2–3)	-0.383	-1.264	-0.837
	Calibration slope used for linear predictor correction	-	0.402	0.950
	Deviation from recalibration regression coefficient: WC ≥ 94cm in men and ≥ 80cm in women	-	-	0.890
	Parent or sibling history of diabetes	-	-	-0.253
	BMI ≥ 30 kg/m^2^	-	-	0.115
Rotterdam Predictive model	Correction factor (1) / Calibration intercept (2–3)	0.593	-0.595	0.391
	Calibration slope used for linear predictor correction	-	0.541	1.134
	Deviation from recalibration regression coefficient: Male gender	-	-	-0.783
Simplified Finnish Diabetes Risk model	Correction factor (1) / Calibration intercept (2–3)	1.212	-0.639	-0.256
	Calibration slope used for linear predictor correction	-	0.388	0.874
	Deviation from recalibration regression coefficient: 45 years ≤ age ≤ 54 years	-	-	-0.390
	Prescribed antihypertensive medication	-	-	0.330

Method 1: correction factor updated intercept; Method 2: both the intercept and the regression coefficients of the variables using the intercept and calibration slope from Method 1; Method 3: Extra adjustment of predictors with a different effect in the updating set compared to the derivation set, after recalibration by Method 2

**Table 3:** Intercept and regression coefficients of the updated models per existing model updated.

		Method 0	Method 4	Method 5			Method 0	Method 4	Method 5
Cambridge	Intercept	-6.322	-3.419	-0.905	Rotterdam	Intercept	-3.020	-2.653	-1.435
Diabetes	Female gender	-0.879	-0.540	-	Predictive	Age per 5 years: 55 years to >75	0.190	0.236***	-
Risk model	Use of antihypertensive medication	1.222	0.587**	-	model	Male gender	0.460	0.184	-
	Prescribed steroids	2.191	-0.657	-		Use of antihypertensive medication	0.420	0.664**	-
	Age	0.063	0.030***	-		BMI ≥ 30 kg/m^2^	0.510	0.767***	-
	25 kg/m^2^ ≤ BMI ≥ 27.49 kg/m^2^	0.699	0.291	-		Linear predictor	-	-	0.857
	27.5 kg/m^2^ ≤ BMI ≤ 29.99 kg/m^2^	1.970	0.002	-		Parent and sibling has diabetes	-	-	1.136
	BMI ≥ 30 kg/m^2^	2.518	0.695***	-		WC ≥ 94cm (M) and ≥ 80cm (W)	-	-	1.290
	Parent or sibling has diabetes	0.728	-0.142	-	Simplified	Intercept	-5.514	-4.080	-1.627
	Parent and sibling has diabetes	0.753	0.989*	-	Finnish	45 years ≤ age ≤ 54 years	0.628	0.108	-
	Ex-smoker	-0.218	-11.312	-	Diabetes	55 years ≤ age ≤ 64 years	0.892	0.692	-
	Current smoker	0.855	-0.054	-	Risk model	25 kg/m^2^ ≤ BMI < 30 kg/m^2^	0.165	2.569	-
	Linear predictor	-	-	0.865		BMI > 30 kg/m^2^	1.096	3.075	-
	WC ≥ 94cm (M) and ≥ 80cm (W)	-	-	0.789		94cm ≤ WC < 102cm in men 80cm ≤ WC < 88cm in women	0.857	-0.337*	-
Kuwaiti	Intercept	-5.018	-3.576	-1.204		WC ≥ 102cm (M) and ≥ 88cm (W)	1.350	-1.053**	-
Risk model	Sibling history of diabetes	0.979	0.680**	-		Use of antihypertensive medication	0.711	1.077***	-
	Use of antihypertensive medication	0.978	0.755***	-		History of high blood glucose†	-	-	-
	Age ≥ 35 years	1.315	1.029*	-		Linear predictor	-	-	0.885
	Waist circumference > 100 cm	1.930	0.992***	-		SBP	-	-	0.011
	Linear predictor	-	-	0.955		Parent and sibling has diabetes	-	-	0.956
	SBP	-	-	0.009					
Omani	Intercept	-4.700	-4.716	-	Developed	Intercept	-5.136	-	-
Diabetes	40 years ≤ age ≤ 59 years	1.800	0.941**	-	model	Age in years	0.028***	-	-
Risk model	Age ≥ 60 years	2.300	1.544***	-		Parent and sibling has diabetes	1.058**	-	-
	WC ≥ 94cm (M) and ≥ 80cm (W)	0.380	0.088**	-		WC ≥ 94cm (M) and ≥ 80cm (W)	1.154**	-	-
	25 kg/m^2^ ≤ BMI < 30 kg/m^2^	0.540	1.880	-		Use of antihypertensive medication	0.516**	-	-
	BMI ≥ 30 kg/m^2^	0.690	1.967	-		SBP	0.004	-	-
	Parental or sibling history of diabetes	1.900	0.447	-		Use of lipid lowering medication	-0.146	-	-
	SBP≥140 and/or DBP≥90	0.730	0.529	-		BMI > 30 kg/m^2^	0.281	-	-

Method 0: original risk model; Method 1: correction factor updated intercept; Method 2: both the intercept and the regression coefficients of the variables using the intercept and calibration slope from Method 1; Method 3: Extra adjustment of predictors with a different effect in the updating set compared to the derivation set, after recalibration by Method 2; Method 4: complete re-estimation of the intercept and the regression coefficients, fitting the variables from the original models in the validation dataset; Developed model: stepwise regression with shrinkage to develop to new model.

P-values

* <0.05

** <0.01

*** <0.001.

† assumed to be 0 for all participants due to the nature of this study.

The re-estimation of the models (Method 4) yielded an intercept closer to 0 (when compared to the original model) for all the models, with the exception of the Omani model (-4.700 to -4.716). When comparing the regression coefficients of the variables across the methods for each model, there was variability, with direct comparisons largely difficult due to the differences in predictor categorisation. However, on the large, beta-coefficients were shrunk closer to zero, with BMI and the use of hypertensive medication showing a larger predictive effect in the Omani, Rotterdam and Finnish risk models. Finally, the investigation into the effect of additional predictors showed statistically significant selection in all but the Omani model. A waist circumference of >94 cm in men and > 80 cm in women was added to both the Cambridge and Rotterdam models, systolic blood pressure to both the Kuwaiti and Finnish models, and a parent and sibling history of diabetes to both the Rotterdam and Finnish models. The development of a model in this dataset, while not the aim of this study, included these three predictors, as well as age, the use of hypertensive medication, the use of lipid lowering medication and a BMI > 30 kg/m^2^.

### Model performance

**Fig 1:**
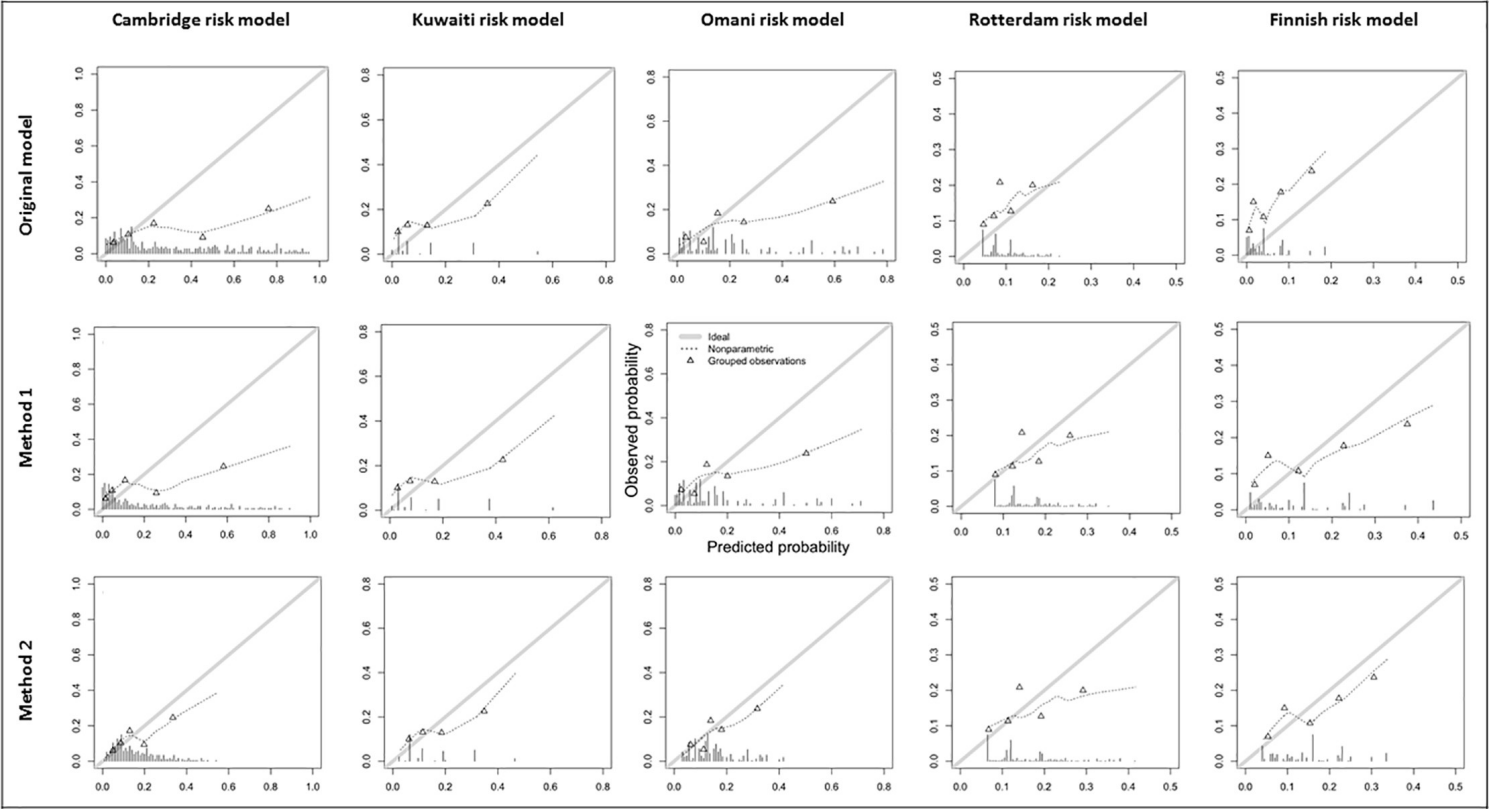
Calibration curves for the risk prediction models across the updating methods.

Tables 4 and 5 show that the model performance across the methods in both the training and test datasets. The original models, fit as they were developed in the full dataset, showed average to moderate discrimination and poor calibration (shown in Fig 1, row 1). As expected, performance across all methods was higher when developing the updated model, with small to large drops in performance when validated. The intercept adjustment and logistic calibration had little effect on the discriminative ability of the models in the training dataset (we expected, and this would have no effect in the full dataset), with a drop in the C-statistic when internally validated. However, calibration was improved across all models, which was largely held after validation, although more pronounced in the Cambridge and Omani models [Method 2, E/O: 0.85 (0.63–1.14) and 0.86 (0.64–1.16), respectively], supported by the calibration curves (Fig 1, row 3) showed a marked improvement closer to the ideal 45° line. Model revision improved the discrimination when developed, however this was only an improvement on previous methods and external validation by the Rotterdam model [C-statistic, Method 1 and 2: 0.58 (0.49–0.67), and Method 3: 0.62 (0.53–0.72)]. The adjustment of the coefficient of a number of predictors in Method 3 did improve the calibration enough to show near perfect E/O ratio’s after internal validation [Omani: 1.01 (0.75–1.36), Rotterdam: 1.03 (0.76–1.38), Finnish: 0.97 (0.72–1.31).

**Table 4:** Performance of the prevalent diabetes risk prediction models across updating methods 0–3.

Models		Reference method	Intercept adjustment (Method 1)		Logistic calibration (Method 2)		Revision (Method 3)	
			Train	Test	Train	Test	Train	Test
Cambridge	E/O (95% CI)	0.48 (0.41; 0.56)	0.79 (0.66; 0.95)	0.67 (0.50–0.90)	1.00 (0.84; 1.20)	0.85 (0.63–1.14)	-	-
Diabetes	Brier score	0.181	0.139	0.147	0.122	0.118	-	-
Risk	Yates slope	0.174	0.153	0.100	0.079	0.049	-	-
Model	C-statistic (95% CI)	0.69 (0.65–0.73)	0.72 (0.67–0.76)	0.62 (0.53–0.71)	0.72 (0.67–0.76)	0.62 (0.53–0.71)	-	-
Kuwaiti Risk	E/O (95% CI)	1.27 (1.09; 1.48)	1.05 (0.88; 1.26)	0.97 (0.72–1.30)	1.00 (0.84; 1.20)	0.91 (0.67–1.22)	-	-
model	Brier score	0.122	0.123	0.123	0.120	0.117	-	-
	Yates slope	0.097	0.127	0.078	0.093	0.055	-	-
	C-statistic (95% CI)	0.70 (0.66–0.74)	0.73 (0.68–0.78)	0.62 (0.53–0.71)	0.73 (0.68–0.78)	0.62 (0.53–0.71)	-	-
Omani	E/O (95% CI)	0.70 (0.60; 0.82)	0.93 (0.78; 1.12)	0.80 (0.59–1.07)	1.00 (0.84; 1.20)	0.86 (0.64–1.16)	1.00 (0.84; 1.20)	1.01 (0.75–1.36)
Diabetes	Brier score	0.142	0.135	0.127	0.125	0.114	0.123	0.114
Risk model	Yates slope	0.110	0.093	0.092	0.052	0.049	0.063	0.036
	C-statistic (95% CI)	0.67 (0.63–0.71)	0.68 (0.63–0.73)	0.65 (0.57–0.73)	0.68 (0.63–0.73)	0.65 (0.57–0.73)	0.70 (0.66–0.70)	0.63 (0.55–0.72)
Rotterdam	E/O (95% CI)	1.62 (1.38; 1.88)	1.01 (0.85; 1.22)	0.88 (0.65–1.18)	1.00 (0.84; 1.20)	0.86 (0.64–1.16)	1.00 (0.84; 1.20)	1.03 (0.76–1.38)
Predictive	Brier score	0.126	0.125	0.118	0.125	0.119	0.122	0.114
model	Yates slope	0.024	0.043	0.017	0.055	0.022	0.071	0.020
	C-statistic (95% CI)	0.66 (0.61–0.70)	0.68 (0.63–0.74)	0.58 (0.49–0.67)	0.68 (0.63–0.74)	0.58 (0.49–0.67)	0.69 (0.64–0.75)	0.62 (0.53–0.72)
Simplified	E/O (95% CI)	2.92 (2.51; 3.41)	1.09 (0.91; 1.31)	1.00 (0.74–1.34)	1.00 (0.84; 1.20)	0.90 (0.67–1.21)	1.00 (0.84; 1.20)	0.97 (0.72–1.31)
Finnish	Brier score	0.133	0.128	0.119	0.125	0.116	0.124	0.115
Diabetes	Yates slope	0.026	0.069	0.050	0.051	0.035	0.061	0.023
Risk model	C-statistic (95% CI)	0.66 (0.62–0.70)	0.68 (0.64–0.73)	0.61 (0.52–0.70)	0.68 (0.64–0.73)	0.61 (0.52–0.70)	0.79 (0.66–0.75)	0.59 (0.50–0.68)

**Table 5:** Performance of the prevalent diabetes risk prediction models across updating methods 4 and 5.

Models		Re-estimation (Method 4)		Addition of new variables (Method 5)	
		Train	Test	Train	Test
Cambridge	E/O (95% CI)	1.00 (0.84; 1.20)	0.89 (0.67–1.20)	1.00 (0.84; 1.20)	1.00 (0.75–1.35)
Diabetes	Brier score	0.127	0.123	0.121	0.114
Risk	Yates slope	0.070	0.030	0.081	0.029
Model	C-statistic (95% CI)	0.66 (0.61–0.72)	0.56 (0.45–0.66)	0.72 (0.68–0.77)	0.63 (0.54–0.72)
Kuwaiti Risk	E/O (95% CI)	1.00 (0.84; 1.20)	0.91 (0.67–1.22)	1.00 (0.84; 1.20)	0.98 (0.73–1.31)
model	Brier score	0.119	0.116	0.118	0.114
	Yates slope	0.090	0.054	0.098	0.031
	C-statistic (95% CI)	0.73 (0.68–0.73)	0.62 (0.53–0.70)	0.74 (0.69–0.74)	0.61 (0.52–0.70)
Omani	E/O (95% CI)	1.00 (0.84; 1.20)	0.88 (0.66–1.19)	-	-
Diabetes	Brier score	0.126	0.119	-	-
Risk model	Yates slope	0.070	0.045	-	-
	C-statistic (95% CI)	0.69 (0.64–0.74)	0.61 (0.52–0.70)	-	-
Rotterdam	E/O (95% CI)	1.00 (0.84; 1.20)	0.87 (0.65–1.17)	1.00 (0.84; 1.20)	0.99 (0.74–1.33)
Predictive	Brier score	0.122	0.118	0.120	0.115
model	Yates slope	0.067	0.033	0.087	0.034
	C-statistic (95% CI)	0.70 (0.65–0.70)	0.60 (0.51–0.69)	0.73 (0.68–0.78)	0.62 (0.53–0.72)
Simplified	E/O (95% CI)	1.00 (0.84; 1.20)	0.88 (0.66–1.18)	1.00 (0.84; 1.20)	0.95 (0.71–1.28)
Finnish	Brier score	0.132	0.125	0.122	0.114
Diabetes	Yates slope	0.058	0.036	0.071	0.033
Risk model	C-statistic (95% CI)	0.64 (0.59–0.69)	0.57 (0.48–0.67)	0.71 (0.66–0.76)	0.63 (0.54–0.72)
Developed	E/O (95% CI)	1.00 (0.84; 1.20)	0.89 (0.67; 1.20)		
Model	Brier score	0.118	0.123		
	Yates slope	0.094	0.031		
	C-statistic (95% CI)	0.74 (0.70–0.79)	0.56 (0.45–0.66)		

Interestingly, the re-estimation of the regression coefficients was not able to increase the validated discrimination or calibration past that achieved by any of the other updating methods for the Cambridge Omani, Rotterdam or Finnish diabetes risk models. The re-estimation of the Kuwaiti model achieved the same results as the basic logistic calibration. Finally, the addition of new predictors resulted in the highest discriminatory and calibration performance combination for all models (not done for the Omani model), when compared to the previous updating methods. The Brier score was slightly reduced with each updating method across all models, but was fairly stable throughout validation. However, the Yates slope was more greatly affected by the updates, decreasing significantly with model validations. The model developed, using no information from an existing model, achieved a developed C-statistic of 0.74 (0.70–0.79), and excellent calibration [E/O: 1.00 (0.84–1.20)], however this performance decreased after validation [C-statistic: 0.56 (0.45–0.66); E/O: 0.89 (0.67–1.20)]. This was in contrast to the higher discriminatory performance of the validated existent models in their original format, however the developed model was better calibrated. Overall, while the original models achieved greater discrimination, the highest performing models, based on both discrimination and calibration, were those updated: the Omani diabetes model following model revision, and the Cambridge diabetes risk model following the addition of waist circumference as a predictor.

## Discussion

The aim of this study was to compare the effects of different updating techniques on the performance of existent diabetes risk prediction models. The performance of the existent models in their original format was not considered sufficient to recommend implementation and the updating methods were intended to aid in bettering the fit of these models. While discrimination was increased when implementing the updating methods, this was lost during internal validation. However, calibration was greatly improved and held following validation. To determine the maximum predictive ability of this population using the available predictors, model development was undertaken, to be used as a comparative. The performance in the development dataset was good, with a number of updating methods matching this performance in development, however overfitting resulted in a 0.18 drop in the c-statistic when internally validated.

The over or under estimated prediction of risk models in new settings may often be due to predictors or characteristics that are not incorporated into the model but do have an effect on the final model parameters. With large disparities between the development and updating populations, as in this study, simple recalibration methods (Methods 1 and 2) are not anticipated to be able to fully adjust for the differences between the development and validation populations. The total re-estimation in the updating dataset (Method 4) is often undertaken in this situation, however revision methods with more simple adjustments (Method 3) may also achieve an increase in performance with the incorporation of this new information in the model. The better performance of the updated Omani (Method 3) and Cambridge (Method 5) diabetes risk prediction models, when compared to the developed model, indicate that the information gained from previously developed models is important to retain. This can be corroborated by the poorer performance of Method 4, where total re-estimation loses the development population information, gained back in Method 5 when the original model is still incorporated.

While higher discrimination and calibration would have been beneficial, it must be noted that this dataset is relatively small, with fewer males, which may have played a role in the performance of the models. This is the first study investigating the performance of prevalent diabetes risk prediction models with updating methods in Africa, and there are a large number of variables collected in the Bellville South cohort database, allowing for five existent diabetes risk prediction models to be validated and updated simultaneously Although unlikely, there may be characteristics of the population which better predict prevalent diabetes, which were not collected.

Model validation and updating is unquestionably advocated to prevent adding models to the already saturated literature. The incorporation of information from a, generally larger and statistically more powerful, development population is important in achieving optimum model prediction. The increase in the external validation of existent models, with attempts to better fit them to a different setting, will allow for the identification of models that are of limited value and the implementation of genuinely useful models, aiding diabetes screening in developing countries where large powerful studies for model development are not as readily available. And while there may be situations where the largely diverse population setting may make existent models possibly too different for even the most complex of updating methods, there is certainly still use for them [5, 6, 23].

In conclusion, comparison of the updating methods employed showed that the more extensive methods incorporating development population information were superior over simpler intercept adjustment or logistic calibration. While updating methods on models validated in empirical data were able to improve calibration, they did not achieve the discrimination of the models in their original format during externally validated. However, the best discrimination and calibration combination was achieved from model updating, over external validation and model development. Unfortunately, the increase in model performance, despite updating methods, was not great enough to recommend further investigation or implementation recommendation.
